# Autophagy-related lncRNAs in tumor progression and drug resistance: A double-edged sword

**DOI:** 10.1016/j.gendis.2023.04.015

**Published:** 2023-06-16

**Authors:** Yunchao Zhang, Jiayu Tang, Cheng Wang, Qinxiu Zhang, Anqi Zeng, Linjiang Song

**Affiliations:** a School of Medical and Life Sciences, Chengdu University of Traditional Chinese Medicine, Chengdu, Sichuan 611137, China; b Institute of Translational Pharmacology and Clinical Application, Sichuan Academy of Chinese Medical Science, Chengdu, Sichuan 610041, China; c School of Pharmacy, Chengdu University of Traditional Chinese Medicine, Chengdu, Sichuan 611137, China

**Keywords:** Apoptosis, Autophagy, Biomarker, Cancer, Drug resistance, LncRNA

## Abstract

The incidence and mortality rates of cancer are increasing every year worldwide but the survival rate of cancer patients is still unsatisfactory. Therefore, it is necessary to further elucidate the molecular mechanisms involved in tumor development and drug resistance to improve cancer cure or survival rates. In recent years, autophagy has become a hot topic in the field of oncology research, which plays a double-edged role in tumorigenesis, progression, and drug resistance. Meanwhile, long non-coding RNA (lncRNA) has also been shown to regulate autophagy, and the two-sided nature of autophagy determines the dual regulatory role of autophagy-related lncRNAs (ARlncRNAs). Therefore, ARlncRNAs can be effective therapeutic targets for various cancers. Furthermore, the high abundance and stability of ARlncRNAs in tumor tissues make them promising biomarkers. In this review, we summarized the roles and mechanisms of ARlncRNAs in tumor cell proliferation, apoptosis, migration, invasion, drug resistance, angiogenesis, radiation resistance, and immune regulation. In addition, we described the clinical significance of these ARlncRNAs, including as biomarkers/therapeutic targets and their association with clinical drugs.

## Introduction

The incidence and fatality rates of cancer are rising year by year.^1^ Notwithstanding a conflation of surgical intervention, chemotherapeutic agents, radiotherapeutic measures, and targeted molecular therapy, the efficacy of these treatments in ameliorating the survival prospects of several cancers remain subpar. Timely detection and intervention can potentially augment the probability of recovery or survival. Consequently, additional molecular network studies are imperative to completely explicate the pathogenic mechanisms underlying the genesis and progression of cancers, as well as drug resistance, and to discern novel biomarkers and therapeutic targets.^2^, ^3^, ^4^, ^5^, ^6^, ^7^, ^8^, ^9^, ^10^

Non-coding RNAs that are longer than 200 nucleotides are called long non-coding RNAs (lncRNAs), which can be involved in the physiopathological processes of various tumors.^11^^,^^12^ Their roles are exerted by several mechanisms involving: i) lncRNAs regulate target genes downstream of miRNAs by competitively adsorbing and down-regulating miRNAs through base complementation. ii) lncRNAs can bind epigenetically related proteins to modify the post-transcriptional translation of specific proteins. iii) lncRNAs can directly bind chromosomal DNA to repress gene transcription or enhance gene transcription by attracting transcription factors. iv) lncRNAs can act as precursors to miRNAs. v) lncRNAs can interact with proteins and affect their activity. vi) lncRNAs can interact with genomic DNA to form triplexes thereby regulating target gene transcription.^13^, ^14^, ^15^, ^16^

There exist three distinct forms of autophagy, namely, macroautophagy, microautophagy, and chaperone-mediated autophagy.^17^, ^18^, ^19^ In our study, the term autophagy pertains specifically to macroautophagy, which is extensively researched and holds a significant functional position.^13^ The generation of autophagosomes encompasses four principal phases, namely, initiation, phagocyte nucleation, elongation and closure of autophagosomes, and fusion of autolysosomes.^20^ These processes involve the participation of several autophagy-related gene (ATG) proteins. Among mammals, there are five core ATG protein subgroups: the Vps34-Beclin 1 class III PI3 kinase complex, Unc-51-like autophagy-activating kinase 1 (ULK1) protein kinase complex, ATG12 coupling system, ATG9-WIPI-1 complex, and the microtubule-associated protein 1 light chain 3 (LC3) coupling system.^21^

**Figure 1:**
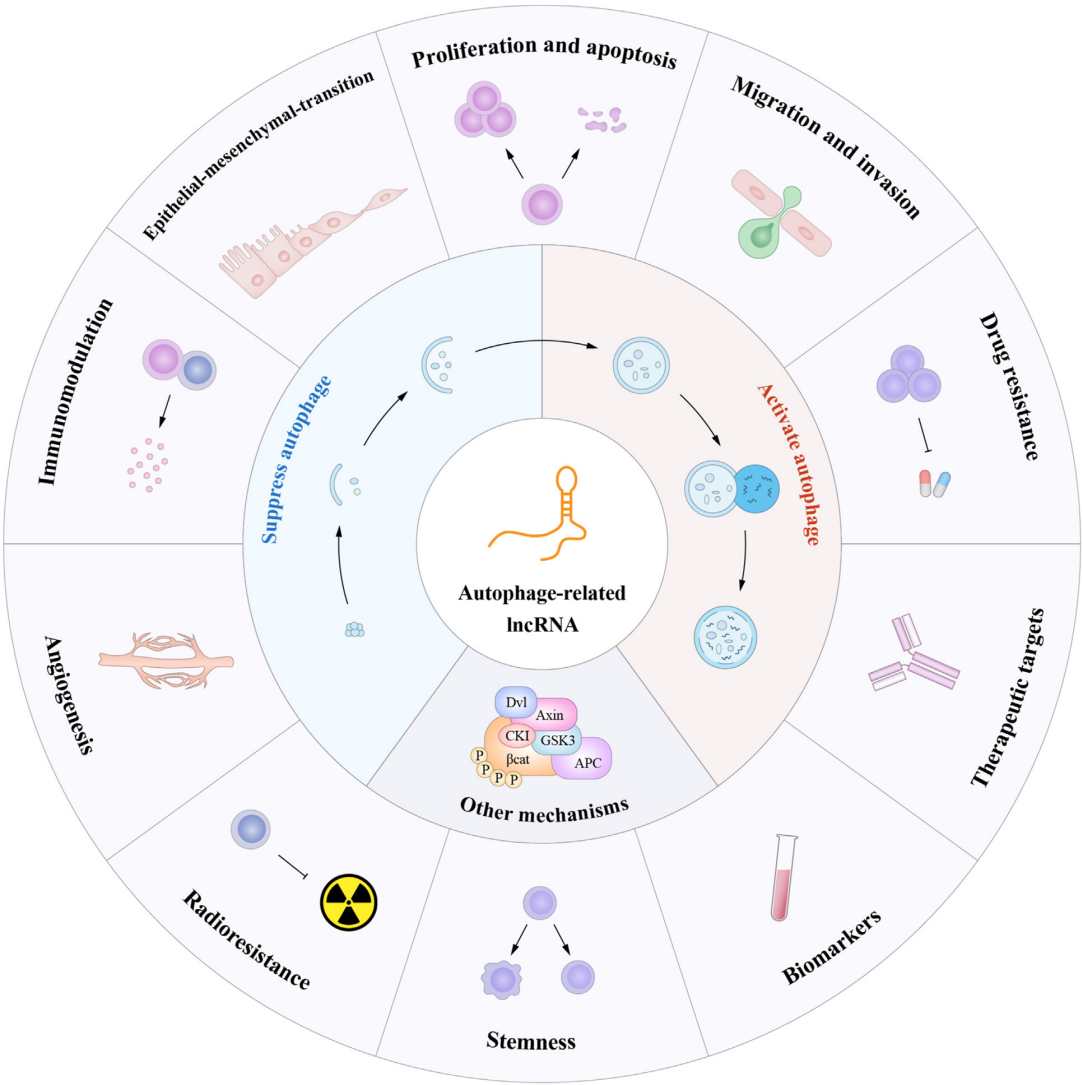
Mechanism, function, and clinical significance of ARlncRNAs.

Interestingly, autophagy plays a double-edged role in tumorigenesis, progression, and drug resistance. During the nascent phases of neoplasms, autophagy exerts an inhibitory effect on tumorigenesis by enzymatically decomposing oncogenic elements; however, as the malignancies advance, autophagy shields the tumor cells by ameliorating the stressful conditions present in the microenvironment of the tumor.^6^^,^^22^ Many complex mechanisms are involved, including the regulation of autophagy-related lncRNAs (ARlncRNAs). The two-sided nature of autophagy determines the dual regulatory role of ARlncRNAs.^17^^,^^23^, ^24^, ^25^, ^26^, ^27^ Thus, ARlncRNAs can serve as effective therapeutic targets for a variety of cancers. Additionally, the high abundance of ARlncRNAs in tumor tissues and their circulating stability make them promising biomarkers for the diagnosis and prognosis of various tumors.^9^^,^^28^ In this review, we summarize the roles and mechanisms of ARlncRNAs in tumor cell proliferation and apoptosis, migration, and invasion, drug resistance, angiogenesis, radioresistance, and immune regulation. In addition, we describe the clinical significance of these ARlncRNAs, including as biomarkers/therapeutic targets and their association with clinical pharmacotherapy (Fig. 1).

## ARlncRNAs promote tumor drug resistance

### ARlncRNAs activate autophagy to promote tumor drug resistance

Numerous ARlncRNAs exhibit up-regulation in diverse carcinomas and foster resistance to chemotherapeutic agents via a variety of mechanisms. The ARlncRNA/miRNA/ATG axis orchestrates the regulation of autophagy-mediated drug resistance, whereby ATG5 serves as a crucial mediator of autophagy. In gastric cancer (GC), the ARlncRNA MALAT1 intensifies cisplatin resistance through the stimulation of autophagy, by down-regulating miR-30b and concomitantly up-regulating ATG5. Unexpectedly, Hu et al found that MALAT1 knockdown exerted no effect on ATG5 mRNA levels in SGC7901/VCR cells.^29^ The discrepancy between the results of these two reports may be due to the intracellular environment.^29^^,^^30^ In contrast, ARlncRNA FEZF1-AS1 specifically targets the up-regulation of ATG5 to increase multidrug resistance in GC.^31^ ATG7 is an E1-like protease that activates the autophagic ubiquitin-like proteins ATG8 and ATG12. ARlncRNA BLACAT1 promotes ATG7 expression via miR-17, thereby promoting autophagy and facilitating chemoresistance in non-small cell lung cancer (NSCLC).^32^ ATG14 can act as a potent activator of autophagy. By competitively connecting to miR-188-3p, EIF3J-DT increases the stability of ATG14 mRNA and inhibits its degradation. Consequently, EIF3J-DT enhances the transcription of ATG14 and leads to activating autophagy and chemoresistance.^33^ The C-terminal peptide of LC3 protein requires cleavage by mammalian ATG4 homologs, thereby promoting double-membrane autophagosome formation. ARlncRNA KCNQ1OT1 targets the miR-34a/ATG4B to enhance oxaliplatin chemoresistance in colon cancer.^34^

ULK1 is an autophagy-associated gene and a target of miRNA. The lncRNA/miRNA/ULK1 axis can regulate autophagy-mediated drug resistance. ARlncRNA LUCAT1 promotes cisplatin resistance in NSCLC via controlling the miR-514a-3p/ULK1.^35^ In colorectal cancer (CRC), the ARlncRNA SNHG6 facilitates resistance to 5-Fluorouracil (5-Fu) by sequestering miR-26a-5p and thus augmenting ULK1-mediated autophagy.^14^ The ARlncRNA Sox2OT-V7 exerts a dual mechanism to up-regulate ULK1 by acting as a miRNA sponge for either miR-142 or miR-204, thereby augmenting autophagy and contributing to drug resistance. Additionally, ULK phosphorylates Beclin-1 after amino acid withdrawal.^21^ Beclin1 is an essential part of autophagosome formation, and up-regulation of Beclin1 activates autophagy. ARlncRNA HOTAIR down-regulates miR-17-5p expression and enhances Beclin1 expression in RC cells, thus promoting cellular autophagy.^36^

Drug resistance is not only affected by autophagy but also regulated by other signaling pathways. Some ARlncRNAs can dual-regulate drug resistance through autophagy and other signaling pathways. The ARlncRNA PVT1 is known to augment gemcitabine resistance in pancreatic cancer by coordinated regulation of the miR-619-5p/Pygo2/Wnt/β-catenin and miR-619-5p/ATG14 pathways. Additionally, there exists a positive feedback loop between the Wnt/β-catenin signal and PVT1 transcription. It remains to be determined whether this positive feedback loop also plays a role in conferring chemoresistance in other types of cancer.^13^ The DNA damage response is a crucial mechanism for preserving genomic stability. ARlncRNA OTUD6B-AS1 increases the formation of paclitaxel resistance in triple-negative breast cancer by inhibiting DNA repair (up-regulating genomic instability) and promoting autophagy. Nonetheless, the mechanism by which metadherin regulates autophagy and DNA damage response-related protein stimulation requires more investigation.^37^

### ARlncRNAs inhibit autophagy to promote tumor drug resistance

Conversely, ARlncRNA HOXA11-AS, ROR can promote drug resistance by inhibiting autophagy and thereby promoting drug resistance. So, they can be used as drugs by themselves or as therapeutic targets to improve the outcome of cancer patients. Researchers performed lentiviral transfection of ovarian cancer cells and showed that HOXA11-AS knockdown significantly inhibited tumor cell motility and promoted cellular autophagy and sensitivity to cisplatin.^38^ The ARlncRNA ROR has been implicated in conferring drug resistance and promoting invasiveness in breast cancer cells.^39^ Further investigations revealed that the inhibition of ROR significantly augmented the sensitivity of breast cancer cells to tamoxifen by promoting autophagy (up-regulation of LC3 and Beclin 1 expression).^40^ The AKT/mammalian target of rapamycin (mTOR) pathway is considered to be an important signal for the regulation of autophagy.^41^ In NSCLC, silencing ROR can reverse cisplatin resistance by inhibiting phosphatidylinositol-3-kinase (PI3K)/protein kinase B (AKT)/mTOR signaling.^42^

## ARlncRNAs inhibit tumor drug resistance

### ARlncRNAs activate autophagy to inhibit tumor drug resistance

These ARlncRNAs that impede drug resistance are frequently found to exhibit low expression levels in drug-resistant tumor tissues. This finding holds significant importance in the selection and application of autophagy-targeted therapeutics. The ARlncRNA EGOT-enhanced autophagy sensitizes paclitaxel cytotoxicity by up-regulating inositol 1,4,5-trisphosphate receptor 1 (ITPR1). Mechanistically, EGOT increases autophagosome accumulation through cis- and trans-up-regulating ITPR1. First, EGOT causes pre-ITPR1 accumulating to up-regulate ITPR1 levels in cis by generating pre-ITPR1/EGOT dsRNA. EGOT engages heterogeneous nuclear ribonucleoproteins to increase alternative splicing of pre-ITPR1 in trans via two binding motifs in fragment 2 of exon 1 of EGOT. Researchers also found that hypoxia can up-regulate EGOT, while estrogen directly inhibits it.^43^ The ARlncRNA GAS5 inhibits tumor growth by inducing autophagy in breast cancer cells. Overexpression of GAS5 up-regulates ULK1/2 in MCF-7 cells without interfering with other autophagy initiation-related proteins or decreasing cell proliferation, migration, or tumor development. The researchers hypothesize that the antitumor effects of GAS5 overexpression are at least partially mediated by autophagy induction. Nevertheless, the mechanism through which GAS5 acts as a negative regulator independent of autophagy is unclear.^44^

### ARlncRNAs inhibit autophagy to inhibit tumor drug resistance

**Figure 2:**
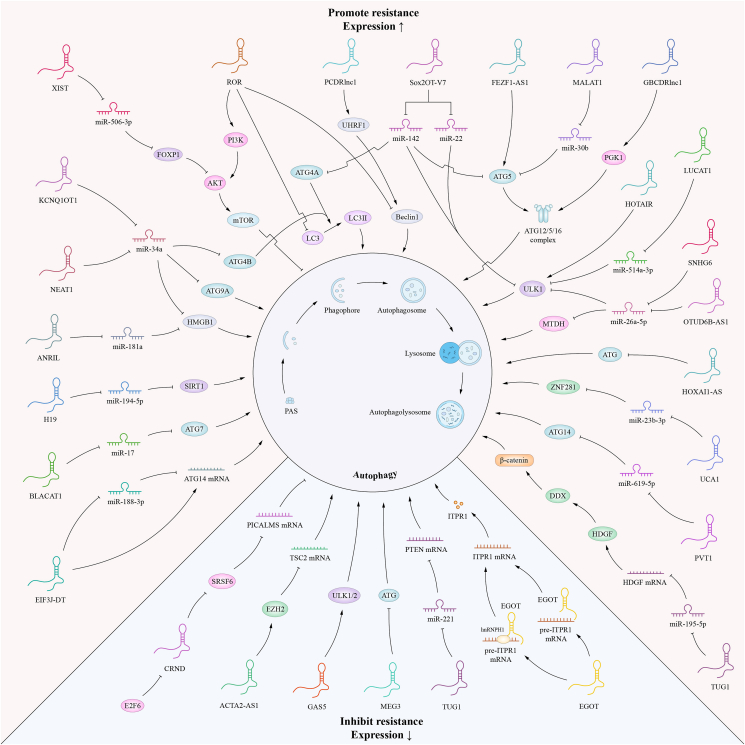
Dual regulatory mechanisms of ARlncRNAs in tumor drug resistance.

The ARlncRNA ACTA2-AS1 suppresses cisplatin resistance in NSCLC via engaging enhancer of Zeste 2 (EZH2) to the TSC2 gene promoter and decreasing tuberous sclerosis complex-2 (TSC2) expression.^45^ By suppressing autophagy, the E2F6-regulated ARlncRNA CRNDE sensitizes GC cells to chemotherapy. Mechanistically, overexpression of CRNDE suppresses autophagy and promotes apoptosis, making GC cells more sensitive to chemotherapeutic treatments. In addition, the classical transcriptional inhibitor E2F6 was shown to up-regulate and suppress CRNDE expression in GC.^46^ CRNDE can also disrupt autophagy-mediated chemoresistance by interacting with and decreasing the protein stability of the downstream target gene serine/arginine-rich splicing factor 6 (SRSF6) and thus decreasing the selective splicing of phosphatidylinositol binding clathrin-assembly protein (PICALM) mRNA.^47^ The ARlncRNA MEG3 has been observed to enhance sensitivity to vincristine by impeding autophagy in the context of lung cancer chemotherapy. Researchers have demonstrated that MEG3 expression was substantially down-regulated in drug-resistant cells compared to non-resistant cells, as seen *in vitro*. As a result, overexpression of MEG3 markedly suppressed the viability and proliferation of both drug-resistant and non-resistant lung cancer cells.^48^ More information and mechanisms of ARlncRNAs regulating tumor drug resistance are shown in Table 1 and Figure 2.

**Table 1:** ARlncRNAs that regulate tumor drug resistance.

lncRNA	Levels	Cancer Type	Autophagy	Mechanisms of Autophagy	Drug	Drug resistance	Reference
EGOT	↓	Breast cancer	Activates	ITPR1 ↑	Paclitaxel	Inhibits	^43^
GAS5	↓	Breast cancer	Activates	ULK1/2↑	Cisplatin	Inhibits	^44^
TUG1	↓	Non-small cell lung cancer	Activates	miR-221/PTEN	Cisplatin	Inhibits	^88^
ACTA2-AS1	↓	Non-small cell lung cancer	Suppresses	Recruites EZH2 to TSC2 gene promoter	Cisplatin	Inhibits	^45^
CRNDE	↓	Gastric cancer	Suppresses	E2F6-CRNDE axis	Oxaliplatin/5-Fu	Inhibits	^46^
MEG3	↓	Lung cancer	Suppresses	–	Vincristine	Inhibits	^48^
CRNDE	↓	Gastric cancer	Suppresses	SRSF6/PICALM	5-FU/oxaliplatin	Inhibits	^47^
H19	↑	Colorectal cancer	Activates	miR-194-5p/SIRT1	5-Fu	Promotes	^89^
NEAT1	↑	Colorectal cancer	Activates	miR-34a/HMGB1/ATG9A/ATG4B	5-Fu	Promotes	^90^
SNHG6	↑	Colorectal cancer	Activates	miR-26a-5p bind to SNHG6 and target ULK1	5-Fu	Promotes	^14^
UCA1	↑	Colorectal cancer	Activates	miR-23b-3p/ZNF281 Axis	5-Fu	Promotes	^91^
XIST	↑	Ovarian cancer	Activates	miR-506-3p/FOXP1/AKT/mTOR	Carboplatin	Promotes	^41^
HULC	↑	Gastric cancer	Activates	FoxM1	Cisplatin	Promotes	^80^
LUCAT1	↑	Non-small cell lung cancer	Activates	miR-514a-3p/ULK1	Cisplatin	Promotes	^35^
MALAT1	↑	Gastric cancer	Activates	microRNA-30b/ATG 5	Cisplatin	Promotes	^29^
TUG1	↑	Colorectal cancer	Activates	miR-195-5p/HDGF/DDX5/β-catenin	Cisplatin	Promotes	^92^
BLACAT1	↑	Non-small cell lung cancer	Activates	miR-17/ATG7	Cisplatin	Promotes	^32^
HOTAIR	↑	Non-small cell lung cancer	Activates	ULK1↑	Crizotinib	Promotes	^93^
FEZF1-AS1	↑	Gastric cancer	Activates	ATG5	5-FU/cisplatin	Promotes	^31^
PCDRlnc1	↑	Prostate cancer	Activates	UHRF1 protein/Beclin-1	Docetaxel	Promotes	^94^
Sox2OT-V7	↑	Osteosarcoma	Activates	miR-142/ULK1, ATG4A, and ATG5 miR-22/ULK1	Doxorubicin	Promotes	^21^
GBCDRlnc1	↑	Gallbladder cancer	Activates	PGK1/ATG5-ATG12 conjugate.	Doxorubicin	Promotes	^20^
MITA1	↑	Non-small-cell lung cancer	Activates	–	Gefitinib	Promotes	^95^
ANRIL	↑	Pancreatic cancer	Activates	miR-181a/HMGB1	Gemcitabine	Promotes	^96^
PVT1	↑	Pancreatic cancer	Activates	miR-619-5p/ATG14	Gemcitabine	Promotes	^13^
HOTAIR	↑	Gastrointestinal stromal tumor	Activates	miR-130a/ATG2B	Imatinib	Promotes	^97^
KCNQ1OT1	↑	Colon cancer	Activates	miR-34a/Atg4B	Oxaliplatin	Promotes	^34^
EIF3J-DT	↑	Gastric cancer	Activates	ATG14	Oxaliplatin/5-Fu	Promotes	^33^
OTUD6B-AS1	↑	Triple negative breast cancer	Activates	miR-26a-5p/MTDH	Paclitaxel	Promotes	^37^
HOTAIR	↑	Renal cancer	Activates	miR-17-5p/Beclin1	Sunitinib	Promotes	^36^
HOXA11-AS	↑	Ovarian cancer	Suppresses	ATG	Cisplatin	Promotes	^38^
ROR	↑	Breast cancer	Suppresses	LC3 and Beclin 1↑	Tamoxifen	Promotes	^40^

## ARlncRNAs promote tumor cell proliferation and inhibit apoptosis

### ARlncRNAs activate autophagy to promote tumor cell proliferation and inhibit apoptosis

Tumor-promoting ARlncRNAs tend to be up-regulated in tissues. The mechanistic target of rapamycin complex 1 (mTORC1) is a major positive modulator of cell proliferation and growth.^49^ Partition-defective 3 (PARD3) is located downstream of mTORC1 and AMP-activated protein kinase (AMPK) and senses amino acid and energy signaling to trigger the initiation of autophagy. ARlncRNA SLCO4A1-AS1 can down-regulate miR-508-3p and thus up-regulate PARD3 expression to promote autophagy and proliferation of CRC cells.^7^ The ARlncRNA MALAT1 can sponge miR-204 in GC to up-regulate transient receptor potential melastatin 3 (TRPM3). TRPM3 has been shown to modulate the expression of LC3A and LC3B, which can activate oncogenic autophagy and facilitate cancer progression. Moreover, the MALAT1/miR-204/LC3B signaling pathway plays a crucial role in H. pylori-induced gastric cancer and is involved in the regulation of autophagy during infection. Further research will focus on elucidating the molecular mechanisms by which this axis regulates autophagy and contributes to H. pylori-induced tumorigenesis.^5^ In addition, MALAT1 activates autophagy to promote cell proliferation and reduce apoptosis by down-regulating miR-101 expression in CRC cell lines.^11^ In multiple myeloma, MALAT-1 increases high mobility group box-1 (HMGB1) to enhance autophagy, hence inhibiting cancer cell death.^50^ In NSCLC patients, a disintegrin and metallopeptidase 28 (ADAM28) has been reported to relate to tumor growth and lymphatic metastasis. ARlncRNA NEAT1 induces autophagy by stimulating the Janus kinase (JAK)/signal transducer and activator of transcription (STAT)3 signaling cascade via the miR-128-3p/ADAM28 axis in NSCLC. However, it could not be determined if ADAM28 directly promotes the activation of the JAK2/STAT3 or indirectly regulates the JAK2/STAT3 by targeting other proteins or RNAs (*e.g.*, lncRNA, miRNA, or circRNA). This is a question that must be investigated in future research.^51^

### ARlncRNAs inhibit autophagy to promote tumor cell proliferation and inhibit apoptosis

Wnt/β-catenin and PI3K/AKT are essential signals that govern cell growth and metastasis. mTOR, which is positioned downstream of the PI3K/AKT, is a negative key controller for autophagy. By regulating EZH2, ARlncRNA SNHG1 stimulates Wnt/β-catenin and PI3K/AKT/mTOR to promote prostate cancer cell growth and inhibit apoptosis and autophagy.^52^ In oral squamous cell carcinoma, overexpression of ARlncRNA CASC9 can suppress autophagy-mediated apoptosis through the AKT/mTOR, hence boosting tumor growth.^53^ The oncogenic factor paired-box 6 (PAX6) of BC belongs to a family of cassette transcription factors that can influence the behavior of tumor cells. The ARlncRNA DANCR/miR-758/3p-PAX6 axis can inhibit autophagy and apoptosis in mammary cancer cells.^8^ Activation of recombinant nuclear factor I/B (NFIB), one of the main proteins affecting tumor cell differentiation, may affect EC progression. ARlncRNA DRAIC/miR-149-5p/NFIB axis can inhibit autophagy and apoptosis in esophageal cancer.^54^ FK506-binding protein 4 (FKBP4) is a member of the pro-immune protein family, and FKBP4 plays a role in immune regulation and essential cellular processes involving protein folding and transport. ARlncRNA MAFG-AS1 can sponge miR-3612 to increase the expression of FKBP4, which activates cell proliferation and inhibits apoptosis and autophagy.^55^

## ARlncRNAs inhibit tumor cell proliferation and promote apoptosis

### ARlncRNAs activate autophagy to inhibit tumor cell proliferation and promote apoptosis

These tumor suppressor ARlncRNAs tend to be down-regulated in tissues. Activating transcription factor 2 (ATF2) is critical in cell development and survival. Researchers found that ATF2 binds to the promoter region of ARlncRNA GAS8-AS1 and activates its expression. GAS8-AS1 could promote papillary thyroid cell autophagy via miR-187-3p/ATG5 and miR-1343-3p/ATG7 thereby inhibiting cancer cell proliferation. However, papillary thyroid patients have various kinds of GAS8-AS1 mutations. Notably, the mutant GAS8-AS1 did not modulate miR-187-3p and miR-1343-3p. The reason for this result may be the difference in secondary structure between the mutant and wild-type GAS8-AS1.^56^,^57^

Nuclear factor-kappaB (NF-κB) is associated with gene transcription in immunity, inflammation, and proliferation. It has been demonstrated that several carcinogens increase proliferation and anti-apoptosis by activating NF-κB. Therefore, NF-κB is anticipated to be a novel cancer therapeutic target. ARlncRNA CASC2 was detected to be lowly expressed in colon cancer cells and inhibited cell viability by down-regulating miR19a and inhibiting the NF-κB/p65, suggesting that CASC2 may be a prospective factor for the therapeutic therapy of colon cancer.^58^ Tripartite motif protein 16 (TRIM16) is a positive transcriptional regulator of retinoic acid receptor β2 in retinoid-treated cancer cells and can act as a tumor suppressor.^59^ CASC2 also causes apoptosis and autophagy in colon cancer by regulating the levels of TRIM16 via miR-214.^60^ Interestingly, CASC2 in NSCLC similarly regulates the miR-214/TRIM16 but results in the inhibition of autophagy and promotion of apoptosis in NSCLC.^59^ This different role of ARlncRNAs in different tumors through the same signaling pathway may be caused by differences in the tumor microenvironment and deserves further investigation.

### ARlncRNAs inhibit autophagy to inhibit tumor cell proliferation and promote apoptosis

**Figure 3:**
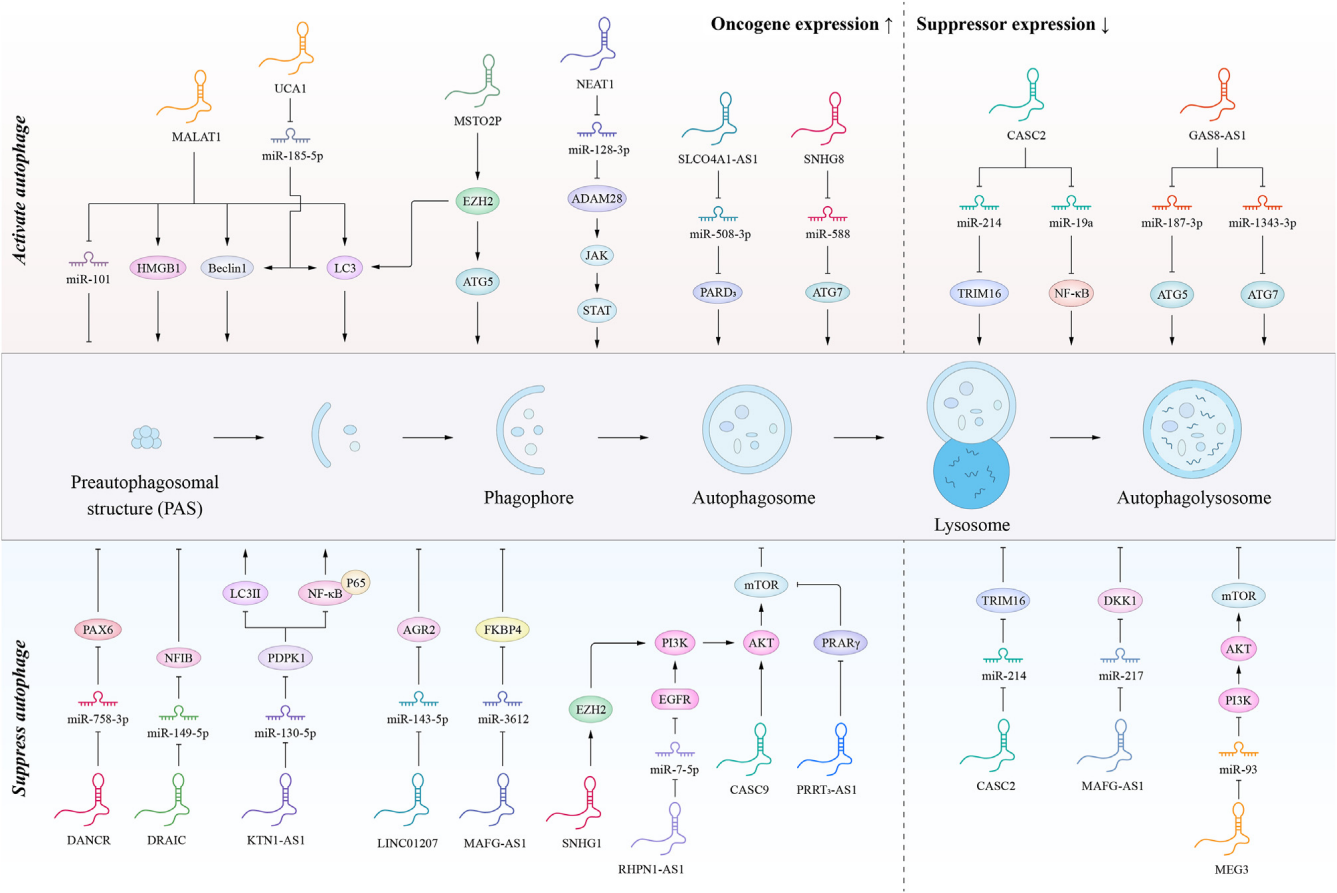
The “double-edged sword” mechanism of ARlncRNAs in tumor proliferation and apoptosis.

The ARlncRNA CLRN1-AS1 acts as a tumor suppressor in pituitary prolactinomas and inhibits autophagy by suppressing the Wnt/β-catenin. Functionally, CLRN1-AS1 inhibits cell proliferation, promotes apoptosis, and suppresses autophagy. Mechanistically, CLRN1-AS1 up-regulates dickkopf WNT signaling pathway inhibitor 1 (DKK1) by sponging miR-217. In addition, they found that the transcriptional repressor upstream of CLRN1-AS1 is Forkhead box P1 (FOXP1).^61^ Similarly, the ARlncRNA MEG3 inhibits autophagy and induces apoptosis by sponging miR-93 to down-regulate the PI3K/AKT/mTOR axis in BC progression.^62^ Interestingly, PTC cells can transmit ARlncRNA SNHG9 to neighboring normal cells via exosomes. Consequently, it can suppress the production of the YBOX3 protein, negatively regulate P21, reduce autophagy, and increase death in normal cells. However, this must be validated in animals and the mechanism by which SNHG9 triggers YBOX3 degradation must be elucidated.^63^ More information and mechanisms of ARlncRNAs regulating tumor proliferation and apoptosis are shown in Table 2 and Figure 3.

**Table 2:** ARlncRNAs that regulate tumor proliferation and apoptosis.

LncRNA	Levels	Cancer Type	Autophagy	Mechanisms of Autophagy	Role	Reference
LCPAT1	↑	Lung cancer	Activates	–	Oncogene	^6^
MALAT1	↑	Gastric cancer	Activates	miR-204↓	Oncogene	^5^
MALAT 1	↑	Colorectal cancer	Activates	miR-101↓	Oncogene	^11^
MALAT-1	↑	Multiple myeloma	Activates	HMGB1↑	Oncogene	^50^
NEAT1	↑	Nonsmall-cell lung cancer	Activates	miR-128-3p/ADAM28	Oncogene	^51^
SLCO4A1-AS1	↑	Colorectal cancer	Activates	miR-508-3p/PARD3	Oncogene	^7^
SNHG8	↑	Colorectal cancer	Activates	miR-588/ATG7	Oncogene	^98^
UCA1	↑	Colorectal cancer	Activates	miR-185-5p-WISP2-Wnt/β-catenin	Oncogene	^99^
MSTO2P	↑	Lung cancer	Activates	EZH2↑	Oncogene	^100^
CASC9	↑	Oral squamous cell carcinoma	Suppresses	AKT/mTOR	Oncogene	^53^
DANCR	↑	Breast cancer	Suppresses	miR-758-3p/PAX6	Oncogene	^8^
DRAIC	↑	Esophageal cancer	Suppresses	miR-149-5p/NFIB	Oncogene	^54^
EGOT	↑	Colon cancer	Suppresses	miR-33a-5p and miR-33b-5p	Oncogene	^9^
KTN1-AS1	↑	Non-small cell lung cancer	Suppresses	miR-130a-5p/PDPK1	Oncogene	^81^
LINC00858	↑	Colon cancer	Suppresses	activates WNK2 promoter methylation	Oncogene	^101^
LINC01207	↑	Pancreatic cancer	Suppresses	miR-143-5p/AGR2	Oncogene	^102^
MAFG-AS1	↑	Breast cancer	Suppresses	miR-3612/FKBP4	Oncogene	^55^
PRRT3-AS1	↑	Prostate cancer	Suppresses	PPARγ/mTOR	Oncogene	^103^
RHPN1-AS1	↑	Prostate cancer	Suppresses	miR-7-5p/EGFR/PI3K/AKT/mTOR	Oncogene	^104^
SNHG1	↑	Prostate cancer	Suppresses	Wnt/β-Catenin and PI3K/AKT/mTOR	Oncogene	^52^
CASC2	↓	Colon cancer	Activates	miR19a↓	Suppressor	^58^
CASC2	↓	Colon cancer	Activates	microRNA-214/TRIM16	Suppressor	^60^
GAS8-AS1	↓	Thyroid Cancer	Activates	miR-187-3p/ATG5 and miR-1343-3p/ATG7	suppressor	^57^
GAS8-AS1	↓	papillary thyroid cancer	Activates	ATG5↑	suppressor	^56^
CASC2	↓	non-small cell lung cancer	Suppresses	miR-214/TRIM16	suppressor	^59^
CLRN1-AS1	↓	pituitary prolactinoma	Suppresses	miR-217/DKK1/Wnt/β-catenin	suppressor	^61^
MEG3	↓	bladder cancer	Suppresses	PI3K/AKT/mTOR	suppressor	^62^

## ARlncRNAs promote tumor migration and invasion

### ARlncRNAs activate autophagy to promote tumor migration and invasion

ARlncRNAs that promote tumor cell migration and invasion are up-regulated in tumor tissues. Matrix infiltration is one of the most important steps in cancer metastasis. Matrix metalloproteinases (MMPs) are a class of zinc-dependent protein hydrolases, and MMP2 mainly hydrolyzes type IV collagen (a key element of the basement membrane) to facilitate cancer cell invasion and spread. Autophagy stimulation by ARlncRNA SNHG1 increases the migration of basal bladder cancer. On the one hand, SNHG1 directly binds to the PP2A catalytic subunit to inhibit its interaction with c-Jun, which then promotes c-Jun phosphorylation and thus mediates MMP2 transcription; on the other hand, SNHG1 initiates autophagy by inducing autophagy-associated protein abundance and further destabilizes miR-34a, thereby decreasing miR- 34a binding to the 3′ UTR of MMP2 mRNA, thus promoting the stabilization of MMP2 mRNA.^64^

The Wnt/β-catenin pathway is recognized as carcinogenic. Functionally, the ARlncRNA SNHG11 promotes cell proliferation, stemness, metastasis, and epithelial–mesenchymal transition in GC via enhancing autophagy. Mechanistically, SNHG11 increases the expression of the transcription of catenin β 1 (CTNNB1) and ATG12 via miR-483-3p/miR-1276 while inhibiting the processing of pre-miR-483/pre-miR-1276. SNHG11 interacts with Cullin 4A (CUL4A) and further stimulates the Wnt/β-catenin pathway by inducing glycogen synthase kinase 3 (GSK-3) ubiquitination. Intriguingly, SNHG11 regulates autophagy via the ATG12 pathway and not the Wnt/β-catenin pathway, but it increases malignant activity in GC cells via both channels.^17^

### ARlncRNAs inhibit autophagy to promote tumor migration and invasion

ARlncRNA CDKN2B-AS1 enhances hepatocellular carcinoma invasion, proliferation, and migration by targeting and inhibiting miR-199a-5p to suppress apoptosis and autophagy, which is expected to be a breakthrough for future diagnosis and treatment. However, the number of patients included in this study was small, and only blood samples were obtained from liver cancer patients for testing. More *in vivo* and *ex vivo* experiments are needed for validation in the future.^65^ STAT3 can induce ARlncRNA HAGLROS overexpression and thus activate mTOR signaling to inhibit autophagy and promote malignant progression in GC cells. HAGLROS regulates mTOR signaling in two ways. On the one hand, HAGLROS can down-regulate miR-100-5p to stimulate mTOR mRNA expression. On the other hand, HAGLROS interacts with mTORC1 to activate mTORC1 signaling to negatively regulate autophagy.^49^ ARlncRNA ADAMTS9-AS1 can also up-regulate mTOR via PI3K/AKT, suppressing the apoptosis and autophagy of bladder cancer cells while boosting their invasion and migration.^66^ In addition, the newly found lncRNA-45 stimulates the mTOR signal to enhance breast tumor progression.^67^

## ARlncRNAs inhibit tumor migration and invasion

### ARlncRNAs activate autophagy to inhibit tumor migration and invasion

These ARlncRNAs that inhibit tumor migration and invasion tend to be up-regulated in expression. Beclin1 belongs to the first autophagy genes discovered in mammals, and it primarily governs autophagosome formation by building complexes with PI3K. Beclin1 could modulate growth factor signalings, such as the AKT and extracellular signal-regulated kinase routes. ARlncRNA GAS8-AS1 suppresses the development of ovarian cancer by triggering the Beclin1-mediated autophagy pathway.^23^ ARlncRNA PTCSC3 inhibits human oral cancer cell proliferation by inducing LC3B-II and Beclin 1 to promote autophagy and up-regulate apoptosis-associated proteins such as Bax.^68^ ARlncRNA ADAMTS9-AS2 promotes autophagy and inhibits the proliferation, migration, and invasion of hepatocellular carcinoma cells by suppressing the PI3K/AKT/mTOR axis. The phosphorylation of AKT and mTOR, the autophagy protein SQSTM1, and the anti-apoptotic protein Bcl-2 were reduced by ADAMTS9-AS2. In contrast, ADAMTS9-AS2 elevated autophagy protein LC3-II, pro-apoptotic protein Bax, and the autophagy regulator Beclin 1. However, this experiment was only conducted on two cell lines; therefore, its experimental outcomes are limited. Further work is required to determine whether the effects of ADAMTS9 on various cellular characteristics are attributable to its impact on the AKT/mTOR.^69^

### ARlncRNAs inhibit autophagy to inhibit tumor migration and invasion

**Figure 4:**
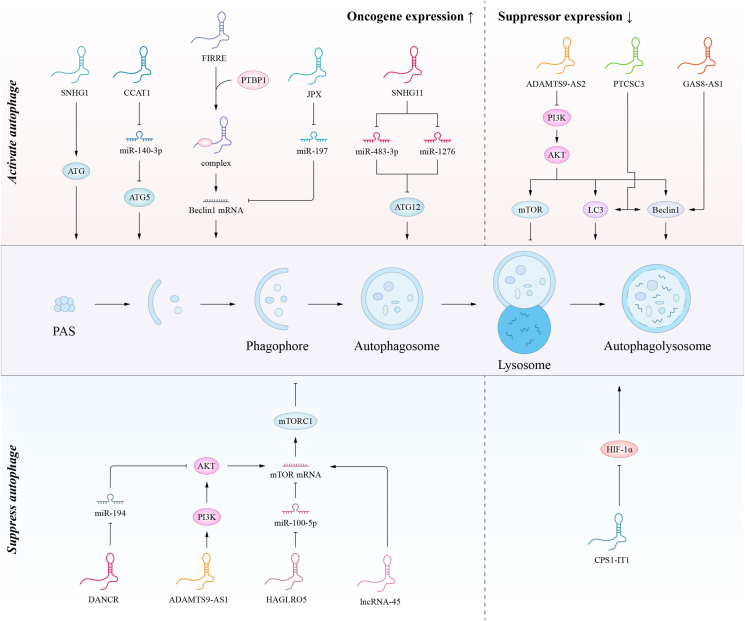
The “double-edged sword” mechanism of ARlncRNAs in tumor migration and invasion.

A study found that ARlncRNA CPS1-IT1 inhibited the growth and metastasis of hepatocellular carcinoma by reducing HIF-1α activation and inhibiting epithelial–mesenchymal transition.^70^ Similarly, one study found that ARlncRNA CPS1-IT1 also inhibits cell proliferation, invasion, and metastasis in colorectal cancer.^71^ Its intrinsic mechanism is related to the phenomenon of hypoxia in the solid tumor microenvironment. Hypoxia induces autophagy through the HIF-1α signaling pathway and promotes tumor cell invasion and metastasis. However, ARlncRNA CPS1-IT1 inhibits hypoxia-induced autophagy by suppressing HIF-1α, thereby suppressing epithelial–mesenchymal transition and CRC metastasis.^72^ More information and mechanisms of ARlncRNAs regulating tumor migration and invasion are shown in Table 3 and Figure 4.

**Table 3:** ARlncRNAs that regulate tumor migration and invasion.

LncRNA	Levels	Cancer type	Autophagy	Mechanisms	Role	Reference
CCAT1	↑	Gastric cancer	Activates	miR-140-3p/ATG5	Oncogene	^105^
FIRRE	↑	Colorectal cancer	Activates	PTBP1	Oncogene	^25^
LCPAT1	↑	Lung cancer	Activates	RCC2	Oncogene	^106^
loc146880	↑	Lung cancer	Activates	ROS	Oncogene	^24^
SNHG1	↑	Basal bladder cancer	Activates	ATG↑	Oncogene	^64^
SNHG11	↑	Gastric cancer	Activates	miR-483-3p/ATG12 and miR-1276/ATG12	Oncogene	^17^
JPX	↑	Gastric cancer	Activates	inhibiting miR-197	Oncogene	^107^
ADAMTS9-AS1	↑	Bladder cancer	Suppresses	activation of PI3K/AKT/mTOR	Oncogene	^66^
CDKN2B-AS1	↑	Liver cancer	Suppresses	miR-199a-5p	Oncogene	^65^
DANCR	↑	Gastric cancer	Suppresses	miR-194/AKT2 axis	Oncogene	^108^
HAGLROS	↑	Gastric cancer	Suppresses	miR-100-5p/mTOR	Oncogene	^49^
lncRNA-45	↑	Breast cancer	Suppresses	activating the mTOR signaling pathway	Oncogene	^67^
ADAMTS9-AS2	↓	Liver cancer	Activates	PI3K/AKT/mTOR	Suppressesor	^69^
GAS8-AS1	↓	Ovarian cancer	Activates	binding with Beclin1	Suppressesor	^23^
PTCSC3	↓	Oral cancer	Activates	LC3B-I/Beclin 1	Suppressesor	^68^
CPS1-IT1	↓	Colorectal cancer	Suppresses	inactivation of HIF-1α	Suppressesor	^72^

## Other regulatory functions of ARlncRNAs

Radiotherapy is the cornerstone of treatment for many types of cancer. However, radiotherapy resistance largely affects the effectiveness of radiotherapy. ARlncRNAs such as HULC and HOTAIR have been found to mediate autophagy and thus promote radiotherapy resistance. Interestingly, HULC binds to Beclin-1, thereby inhibiting Beclin-1 phosphorylation, leading to the inhibition of autophagy through the mTOR pathway to promote radiotherapy resistance. In contrast, HOTAIR promotes autophagy by regulating the miR-93/ATG12 axis in CRC, thereby enhancing radiation resistance. Since they are both up-regulated after radiotherapy, decreased tumor cell survival and cell cycle, and increased apoptosis and radiosensitivity were observed after their knockdown.^73^^,^^74^

Tumor formation and development cannot be separated from angiogenesis and glucose utilization. The highly enriched exosomal ARlncRNA OIP5-AS1 can promote angiogenesis and autophagy in osteosarcomas via miR-153/ATG5. The intrinsic mechanism is that ATG5 regulates angiogenesis in vascular endothelial cells via reactive oxygen species-dependent signal.^26^ Glycolysis is the main pathway of glucose utilization by cancer cells. MYC mRNA is one of the core regulators of glycolysis, which is regulated by the upstream IGF2BP2. Wang et al found that the ARlncRNA LINRIS binds to the ubiquitination site of IGF2BP2, thereby preventing the degradation of IGF2BP2 via the ubiquitination-autophagy pathway.^75^ As a result, its downstream MYC mRNA was stabilized. *In vivo* experiments demonstrated that LINRIS knockdown decreased tumor development in orthotopic and patient-derived xenograft models.^75^

Immune cells and immune-related mediators are essential in tumor growth in the tumor microenvironment. Cancer-associated fibroblasts are a major source of chemicals released by the tumor microenvironment that drive cancer cell growth. Similarly, variations of inflammatory mediators in cancer cells inside the tumor microenvironment result in the transformation of normal fibroblasts into cancer-associated fibroblasts. MALAT1 was found to decrease autophagic flux and increase IL-6 by regulating the PTEN/AKT/mTOR and SQSTM1/NF-κB, thereby transforming fibroblasts into cancer-associated fibroblasts to accelerate GC development. Nevertheless, the mechanism behind IL-6-induced cancer-associated fibroblast activation needs further investigation.^76^ Through pattern recognition receptors such as Toll-like receptors and advanced glycosylation end-product-specific receptors (AGERs), carcinogens can activate the innate immune system. AGERs recognize endogenous molecules released during chronic inflammation and recruit epithelial cells for innate immune cell recruitment. Investigators found that up-regulation of ARlncRNA AGER inhibited lung cancer cell proliferation and migration, induced cell cycle arrest in the G0/G1 phases, and promoted apoptosis. ARlncRNA AGER may also promote cytotoxic activity and autophagy of immune effector cells by up-regulating AGER by sponging miR-185. Thus, it is a potential target for tumor immunotherapy.^27^

## The clinical significance of ARlncRNAs

### ARlncRNAs as biomarkers

Most current cancer biomarkers are proteins or peptides whose changes in tissue or blood levels reflect cancer progression. However, these biomarkers suffer from shortcomings. On the one hand, they produce a large number of false-positive and/or false-negative results, which is partly due to the nature of the biomarker itself. On the other hand, the invasive and inconvenient nature of tissue biopsy hinders its application. Therefore, it is necessary to develop highly sensitive, non-invasive non-protein biomarkers. Due to their great stability and resistance to nuclease-mediated degradation, circulating lncRNAs appear to be more dependable than other circulating nucleic acids.^9^^,^^28^

ARlncRNA CASC9 was substantially expressed in oral squamous cell carcinoma and significantly linked with tumor volume, local lymph node migration, stage of the disease, and overall survival. Therefore, CASC9 could be a potential marker for the diagnosis and prognosis of oral squamous cell carcinoma.^53^ ARlncRNA EGOT is also significantly expressed in colon cancer patients. Survival time is decreased in patients with high EGOT expression. After EGOT overexpression, the apoptosis rate decreased and the cell growth invasion increased. Moreover, EGOT was found to potentially impede the proliferation and spread of colon cancer via modulating autophagy, making it a potential therapeutic target and diagnostic marker for colon cancer.^9^ In addition, due to its involvement in controlling cancer cell resistance to paclitaxel, EGOT might even function as a promising biomarker of paclitaxel response.^43^

### ARlncRNAs are used to construct prognostic models

Due to the low sensitivity or specificity of various circulating lncRNAs for specific cancer types, the diagnostic accuracy of a number of circulating lncRNAs is quite poor when analyzed individually. It has been reported that a single circulating lncRNA does not outperform the diagnostic capabilities of models consisting of several lncRNAs.^28^ Therefore, with the development of bioinformatics, more and more prognostic models are being developed. Prediction models based on lncRNAs and clinical characteristics can effectively predict osteosarcoma.^77^ Hang et al constructed an alternative marker for lung adenocarcinoma consisting of 14 ARlncRNAs and accurately predicted survival, tumor immune microenvironment status, and even the efficacy of immune checkpoint inhibitors in patients with lung adenocarcinoma.^78^ The group with the lowest risk had a higher survival benefit. High-risk scores were negatively connected with abundant peritumor immune cells and stromal cells, as well as a high mutational burden in the tumor. Low-risk patients showed greater PD-1 and CTLA-4 expression and immune checkpoint inhibitors were more effective. Li et al constructed a risk score model based on 18 ARlncRNAs to assess osteosarcoma in breast cancer patients.^79^ They also found that these lncRNAs are participating in the modulation of oxidative phosphorylation, nucleotide excision repair, TGF-β signaling pathway, and multicellular biomolecule metabolism.

### ARlncRNAs as promising targets for tumor therapy

Knockdown of proto-oncogene can promote apoptosis and drug sensitivity of tumor cells and inhibit proliferation and metastasis of tumor cells. In an *in vitro* study, the investigators observed that the p-AKT, p-mTOR, and BCL-2 levels were reduced, while the LC3BII/LC3BI ratio was elevated in oral squamous cells with knockdown of ARlncRNA CASC9. These results suggest that CASC9 induces autophagy and death while suppressing cell proliferation.^53^ In another *in vitro* study, hepatocellular carcinoma cells exhibited weaker viability, invasive and migratory activities, and stronger apoptotic activity after silencing ARlncRNA CDKN2B-AS1 and up-regulating miR-199a-5p.^65^ In *in vivo* studies, inhibition of tumor cell growth was observed after the knockdown of ARlncRNA MALAT-1, HULC, DANCR, and LCPAT1.^6^^,^^50^^,^^80^^,^^81^ By the same token, up-regulation of oncogenes is also an effective anti-tumor strategy. Overexpression of ARlncRNA PTCSC3 leads to a substantial reduction in human oral cancer cell proliferation through the induction of apoptotic cell death^68^ Fang et al constructed the ARlncRNA GAS8-AS1 plasmid to transfect ovarian cancer cells.^23^ After transfection, GAS8-AS1 was up-regulated and inhibited the migratory ability of ovarian cancer cells; in addition, ovarian cancer cells were reduced by GAS8-AS1.

### The connection between clinical pharmacotherapy and ARlncRNAs

Scientists have discerned several pharmacological agents that modulate ARlncRNA and thereby impede tumorigenesis via multiple signaling pathways. These recent findings regarding drug interventions furnish a foundation for novel antineoplastic therapeutic approaches.

Some antitumor drugs exert antitumor effects while up-regulating certain ARlncRNAs that activate autophagy, which reduces the efficacy of the drugs. Therefore, knocking down the ARlncRNA up-regulation induced by these drugs could exert synergistic antitumor effects. The accumulation of misfolded proteins in the endoplasmic reticulum and the activation of unfolded protein responses may lead to endoplasmic reticulum stress. Continuous or exacerbated endoplasmic reticulum stress can lead tumor cells toward apoptosis.^82^ However, endoplasmic reticulum stress can be mitigated by autophagy to preserve its homeostasis.^83^ Researchers found that resveratrol promotes ARlncRNA H19 expression, thereby activating autophagy. Interestingly, knocking down H19 in resveratrol-lowered cells further promoted the impacts of resveratrol on apoptosis, endoplasmic reticulum stress, and cell cycle S-phase arrest, and inhibited cell migration. Therefore, knocking down H19 reverses the resistance of tumor cells to resveratrol treatment.^82^ MALAT1 expression is up-regulated after metformin use in breast cancer, which also implies activation of autophagy. The elevated endoplasmic reticulum stress induced by metformin treatment was further up-regulated after the knockdown of MALAT1 was made. It is suggested that the combination of metformin and MALAT1 knockdown could potentially result in synergistic induction of cellular death.^83^ Ferroptosis is a type of necrotic cell death that is dependent on iron and is characterized by the occurrence of oxidative damage to phospholipids and an up-regulated expression of unsaturated fatty acids in the cellular membrane. This ultimately results in the formation of lipid peroxidation and a subsequent disturbance in the structure of the membrane. Researchers discovered that metformin could trigger ferroptosis by blocking H19-mediated autophagy in breast cancer cells.^84^

Furthermore, it is worth noting that certain antitumor drugs have been found to possess the ability to down-regulate ARlncRNA and thereby exhibit antitumor properties. The administration of propofol was observed to promote apoptosis and decrease autophagic activity by inhibiting the MALAT1/miR-30e/ATG5 axis. *In vivo*, co-treatment with propofol and cisplatin resulted in a marked reduction in both the size and weight of tumors in a GC xenograft model.^85^ Apatinib inhibits cancer stem cell properties and malignant biological behavior of breast cancer stem cells by blocking the Wnt/β-catenin signal through down-regulation of ARlncRNA ROR.^86^ In addition, cisplatin-induced autophagy in HO8910 ovarian cancer cells. Besides, ARlncRNA RP11-135L22.1 overexpression inhibited cisplatin-induced autophagy, thus enhancing the effect of cisplatin on ovarian cancer cells. The combination of cisplatin and RP11-135L22.1 could decrease autophagy, enhance apoptosis, and suppress the activity of ovarian cancer cells.^87^

## Conclusions and perspectives

In recent years, autophagy has become a hot topic in the field of tumor research. However, the two-sided nature of autophagy in tumors has led to the dilemma of targeting autophagy for therapy. Interestingly, ARlncRNAs can mediate both sides of autophagy, thus positively or negatively influencing tumorigenesis, progression, and drug resistance. The dualistic nature of ARlncRNA can be attributed to the heterogeneity of tumor microenvironments, as well as the varied mechanisms of action among different ARlncRNAs. Consequently, these factors must be taken into account when considering targeted therapies. Additionally, a deeper investigation into the regulatory mechanisms governing ARlncRNA-mediated molecular networks is warranted.

Numerous investigations have demonstrated that ARlncRNAs function by modulating specific miRNAs that are downstream of them. Nevertheless, a single lncRNA is not restricted to regulating solely one miRNA, and thus, more extensive exploration of the numerous targets or signaling pathways that lie downstream of ARlncRNAs is indispensable. For example, ATG5-mediated autophagy also involves NF-κB and p53/Rb signaling pathways. Whether ARlncRNAs regulate these pathways requires further study and discussion. Similarly, tumor cell proliferation apoptosis, migration and invasion, and drug resistance are not only affected by autophagy, but some ARlncRNAs can achieve multiple regulations of tumors through autophagy and other non-autophagic signaling pathways. On the one hand, it is necessary to clarify the regulatory mechanisms of these non-autophagy; on the other hand, it is necessary to clarify the crosstalk between these non-autophagy mechanisms and autophagy, which may rescue the failure of autophagy-targeted therapy. For example, the relationship between Wnt/β-catenin and autophagy is complex and controversial and deserves further investigation. In addition, autophagy can promote immune escape during cancer cell development. How the regulation of autophagy in tumor cells by ARlncRNA alters the mechanism of immune system monitoring of tumor cells also deserves further analysis.

Early diagnosis can help improve cancer cure or survival rates. The advantages of ARlncRNA, such as its high stability and relative abundance in circulation, make it a reliable diagnostic and prognostic biomarker. However, the premise is that it needs to be improved; otherwise, there is still the problem of being degraded in circulation. Employing a panel of various ARlncRNAs in model construction may substantially amplify the diagnostic and predictive capabilities. While the study of circulating lncRNA biomarkers is still in its nascent stage, more comprehensive investigations are warranted to uncover circulating biomarkers that can reliably detect cancer in its incipient stages.

In conclusion, ARlncRNAs in tumors are a double-edged sword in mediating tumor development and drug resistance. They mediate autophagy while also affecting tumor cell proliferation apoptosis, migration, and invasion, drug resistance, angiogenesis, glycolysis, radiation therapy resistance, and immune microenvironment; therefore, they can serve as ideal therapeutic targets and biomarkers. However, further studies are needed to elucidate these molecular mechanisms and the crosstalk between them.

## Author contributions

LS, AZ, and QZ contributed to the study's conception. YZ, JT, and CW wrote the manuscript. LS, AZ, and QZ critically revised the manuscript. All authors read and approved the submitted version of the manuscript.

## Conflict of interests

The authors declare that there is no conflict of interests.

## Funding

The work is supported by the Xinglin project of Chengdu University of Traditional Chinese Medicine, Sichuan, China (No. ZKYY2019, MPRC2021012).
